# A Rare Cause of Chest Pain Identified on Point-of-care Echocardiography: A Case Report

**DOI:** 10.5811/cpcem.2021.9.53553

**Published:** 2022-03-07

**Authors:** Brian W. Chin, Kassandra A. King, Nicholas K. George, Michael M. Neeki, Jamshid T. Mistry

**Affiliations:** * Arrowhead Regional Medical Center, Department of Emergency Medicine, Colton, California; † Arrowhead Regional Medical Center, Department of Medicine, Colton, California; ‡ Riverside Community Hospital, Department of Critical Care, Riverside, California

**Keywords:** ultrasound, point-of-care, cardiac mass, case report, chest pain

## Abstract

**Introduction:**

Cardiac masses are a rare cause of chest pain. They can often be missed on a chest radiograph performed to evaluate non-specific chest pain and are not readily evaluated with traditional laboratory testing. However, these masses can be visualized with point-of-care ultrasound.

**Case Report:**

We present a case of a 19-year-old female presenting with intermittent chest pain, palpitations, and weakness present for two months. The patient had previously been evaluated at our emergency department one week earlier and was diagnosed with anxiety before being discharged. Besides a tachycardic and labile heart rate, physical examination and laboratory testing were unremarkable. Point-of-care cardiac echocardiography subsequently demonstrated findings concerning for a cardiac mass.

**Conclusion:**

Cardiac masses are a rare cause of chest pain and palpitations that are easily missed. The advent of point-of-care ultrasonography has afforded us the ability to rapidly assess for structural and functional cardiac abnormalities at bedside, and incorporation of this tool into the evaluation of patients with chest pain offers the ability to detect these rare pathologies.

## INTRODUCTION

The incidence of primary cardiac tumors in the general population is unclear due to rarity of the condition, with most sources citing numbers less than 0.1%.^1^ Secondary tumors from other sites are much more prevalent and outnumber primary tumors by about 20:1. Cardiac pseudotumors such as intracardiac thrombi are also significantly more common than primary cardiac tumors.^2^ Primary cardiac tumors are mostly benign with malignant tumors making up about 10% of all primary cardiac tumors. Of these malignant tumors, sarcomas are the most common. Overall, benign primary cardiac tumors have a good prognosis with most patient responding favorably to surgery while malignant tumors carry a significantly worse prognosis.^3^,^4^,^5^

Symptomatic primary cardiac tumors can cause a range of symptoms including chest pain, arrhythmia, and even sudden death.^6^,^7^,^8^ Additionally, primary tumors can serve as a nidus for the formation of thrombi that can subsequently embolize.^9^ Evaluation of cardiac masses often begins with either transthoracic or transesophageal echocardiography. Cardiac computed tomography and cardiac magnetic resonance imaging can subsequently be obtained if further characterization of the mass is deemed to be necessary.^10^,^11^ Overall, benign primary cardiac tumors have a good prognosis with most patients responding favorably to surgery.^5^

## CASE REPORT

A 19-year-old female presented to the emergency department (ED) with the chief complaint of chest pain, palpitations, and generalized weakness intermittently, which were present for two months but had become more prominent during the prior week and worsening over the immediately preceding day. She had been seen in our ED one week prior for similar symptoms and was diagnosed with anxiety before being discharged. The patient did not report any other previous medical or surgical history, denied any history of familial disease to the best of her knowledge, and reported marijuana use several days earlier.

On presentation, the patient was tachycardic with a rate of 126 beats per minute (bpm), hypertensive at 169/84 millimeters of mercury, and mildly tachypneic with a respiratory rate of 17 breaths per minute. Otherwise, she was afebrile and not hypoxic. Examination revealed a young female who appeared stated age and mildly anxious. Her cardiac exam revealed tachycardia with a regular rhythm and no appreciable murmurs. The patient’s lungs were clear to auscultation bilaterally and she was in no respiratory distress. Her skin was without any notable lesions or rashes, and she had no lower extremity edema or calf tenderness to palpation. Of note, the patient exhibited pronounced heart rate lability and would often be noted to have a resting heart rate between 90–100 bpm on telemetry when alone in the room, which increased to 120–140 bpm whenever staff entered.

**Image 1:**
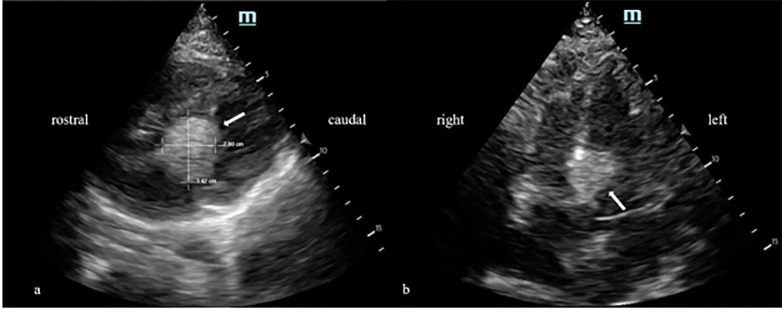
A) Parasternal long view demonstrating hyperechoic cardiac mass measuring 3.42 x 2.80 centimeters (arrow). B) Apical four-chamber view demonstrating same hyperechoic mass on septum (arrow).

**Image 2:**
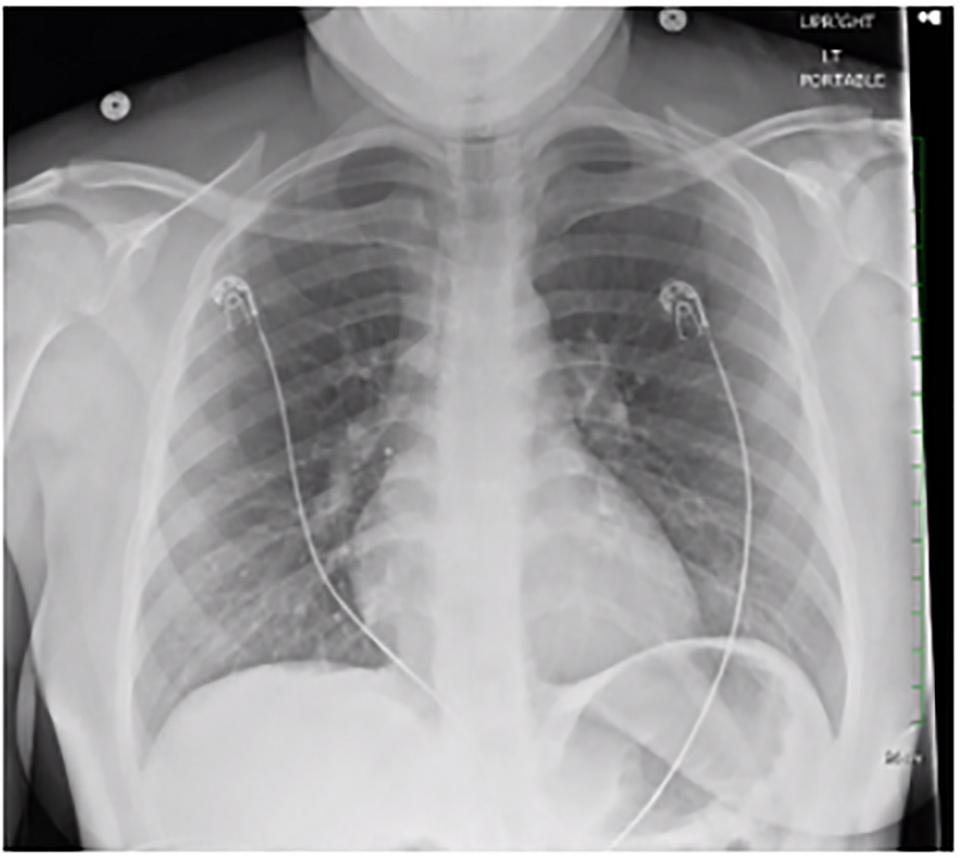
Portable upright anterior-posterior chest radiograph not demonstrating appreciable cardiopulmonary disease.

Point-of-care echocardiography demonstrated an approximately 3.42 x 2.80 centimeters (cm) ovular echogenic structure in the left ventricular outflow tract in the parasternal long window. The structure was also visualized in the apical four-chamber window (Image 1, video). On bedside echocardiography the patient was noted to be tachycardic without global left ventricular hypokinesis, and the right ventricle was not dilated. A chest radiograph was obtained that did not demonstrate any cardiopulmonary abnormality (Image 2), and 12-lead electrocardiogram demonstrated sinus tachycardia without ST-segment changes.

Laboratory testing demonstrated a mild microcytic anemia with a hemoglobin level of 10.6 grams per deciliter (g/dL) (reference range: 11.5–15.5 g/dL) and mean corpuscular volume of 77 femtoliters (fL) (80–100 fL). Otherwise, the patient’s white blood cell counts were within reference range, but she had a mild thrombocytosis at 400 thousand cells per microliter (th/μL) (120–360 th/μL). Chemistries were primarily remarkable for a mildly low potassium level of 3.0 milliequivalents per liter (mEq/L) (3.5–5.5 mEq/L), carbon dioxide level of 19 millimoles per liter (mmol/L) (24–34 mmol/L), iron level of 22 micrograms per deciliter (mcg/dL) (40–150 mcg/dL), and ferritin of 11.6 nanograms per milliliter (ng/mL) (20–300 ng/mL). Urine toxicology returned positive for marijuana, and thyroid stimulating hormone (TSH) levels were within normal reference range. Urinalysis was unremarkable, and urine pregnancy test was negative.

**CPC-EM Capsule**

What do we already know about this clinical entity?

*Cardiac tumors are rare and oftentimes asymptomatic. They are not easily seen on laboratory testing or radiography and are easily missed*.

What makes this presentation of disease reportable?

*This case highlights how point-of-care ultrasonography was able to detect a rare clinical entity that had been missed on previous visits*.

What is the major learning point?

*Ultrasonography should be routinely considered for evaluation of chest pain and can detect several clinical entities not readily captured by other methods*.

How might this improve emergency medicine practice?

*Point-of-care ultrasonography is becoming a staple of emergency medicine. Regular use can help avoid missed diagnoses and delays in treatment*.

The patient received two liters of normal saline for fluid resuscitation and repeat vitals after administration of fluid demonstrated improvement in tachycardia. As seen in the lab values, the patient was mildly anemic with a microcytic anemia consistent with iron deficiency anemia. Unfortunately, date of last menstrual period was unknown; however, this finding is not unexpected secondary to menses in a reproductive-age woman. There was less suspicion for thyroid dysfunction given normal TSH levels, and the patient’s symptoms were considered unlikely to be secondary to acute marijuana toxicity given the reported timeline of use. Urine fractionated metanephrines and plasma free metanephrine levels were not obtained, however, pheochromocytoma should also have been considered in this young, hypertensive patient.

The case was discussed with cardiology, and heparin infusion was initiated over concerns of adherent thrombi that could embolize. The patient was subsequently admitted to the hospital, and a computed tomography (CT) of the thorax with intravenous contrast was performed; however, the CT was not obtained using a cardiac protocol. No mass was noted on the final report. The patient underwent transesophageal echocardiography that confirmed the presence of a 3.5 x 2.25 cm interventricular septal mass just below the aortic valve. The patient was subsequently transferred to a tertiary medical center for cardiothoracic surgical evaluation.

## DISCUSSION

The differential diagnosis for a young female presenting with a chief complaint of chest pain is broad, but with point-of-care ultrasonography we were able to rapidly identify a cause: cardiac mass. The mass did not seem to stem from a valve, and the lack of fever and other Duke criteria for infective endocarditis made endocarditis unlikely.^12^ The patient also did not report any history of immunologic disease, thereby making non-infective vegetations unlikely. Pure thrombus was deemed unlikely given her normal prothrombin time, partial thromboplastin time, international normalized ratio, and location of the mass. Sigmoidal hypertrophic cardiomyopathy could present similarly; however, the general appearance of the mass was more discrete than would be expected with hypertrophy.^13^ In our case, the patient was young and without previous medical history making secondary cardiac tumor, while not impossible, relatively less likely than in the general population. The cardiac mass also appeared to be left-sided in origin, which decreases the likelihood of malignancy since right-sided primary cardiac tumors are more likely to be malignant.^14^ Overall, the mass was most likely a rhabdomyoma, lipoma, or fibroma given ventricular location, although histologic examination would be required for the ultimate diagnosis.^15^

## CONCLUSION

This case demonstrates the benefit of point-of-care echocardiography as an adjunct for chest pain workup. While many patients receive plain films of the chest as part of the workup, this examination notably does not have the ability to assess for structural abnormalities of the heart. As demonstrated by this case, even more advanced imaging modalities such as CT may not detect cardiac masses if a cardiac-specific protocol is not used.^10^ In this regard, ultrasonography can offer improved diagnostic yield when compared to other imaging modalities. Point-of-care ultrasonography holds the unique ability to evaluate for intracardiac pathology at bedside and should routinely be considered for the evaluation of chest pain.

Furthermore, ultrasonography can provide functional insight that traditional imaging often lacks, such as ejection fraction, regurgitant flow across valves, and Doppler flow gradients for obstructive physiology. Although quantification of some of these exams can be technically challenging, even gross qualitative assessments of cardiac structure and function can assist with treatment decisions and resuscitation. In this case, the location of the cardiac mass in the left ventricular outflow tract (LVOT) raised suspicion for obstructive physiology. Additional fluid can help improve cardiac output in intravascularly depleted patients and would be especially helpful for a patient with obstruction from a LVOT mass.

## Supplementary Information

Video Parasternal long axis view visualizing left atrium (LA), left ventricle (LV), aortic root (AR), and right ventricle (RV). White arrow indicates location of mass between aortic root and left ventricle in the left ventricular outflow tract.
